# Patients with degenerative cervical myelopathy exhibit neurophysiological improvement upon extension and flexion: a retrospective cohort study with a minimum 1-year follow-up

**DOI:** 10.1186/s12883-022-02641-1

**Published:** 2022-03-23

**Authors:** Zhengran Yu, Jiacheng Chen, Xing Cheng, Dingxiang Xie, Yuguang Chen, Xuenong Zou, Xinsheng Peng

**Affiliations:** 1 Guangdong Provincial Key Laboratory of Orthopedics and Traumatology, Department of Spine Surgery, The First Affiliated Hospital of Sun Yat-sen University, Guangzhou, 510080 People’s Republic of China; 2 Department of Radiology, The First Affiliated Hospital, Sun Yat-sen University, Guangzhou, China

**Keywords:** Cervical spondylotic myelopathy, Improvement upon extension or flexion, Somatosensory evoked potential, Surgical prognosis, Relevant factors

## Abstract

**Background:**

Cervical extension and flexion are presumably harmful to patients with degenerative cervical myelopathy (DCM) because they worsen medullary compression visible on dynamic magnetic resonance imaging (MRI). Dynamic somatosensory evoked potentials (SSEPs) are an objective tool to measure the electrophysiological function of the spinal cord at different neck positions. In contrast to previous hypotheses, a considerable proportion of patients with DCM present improved SSEPs upon extension and flexion compared to a neutral position.

**Methods:**

Patients with DCM who underwent preoperative dynamic SSEP examinations and subsequent decompression surgeries between 2015 and 2019 were retrospectively evaluated. We compared extension and flexion SSEPs with neutral SSEPs in each patient and classified them into extension-improved (EI) or extension-nonimproved (EN) and flexion-improved (FI) or flexion-nonimproved (FN) groups. Preoperative clinical evaluations, decompression surgical methods and one-year follow-up clinical data were recorded. Cervical spondylolisthesis and cervical alignment types were evaluated on plain cervical lateral radiographs. The number of stenotic segments, Mühle stenosis grade and disc degeneration stage of the most severe segment, and presence of ligamentum flavum hypertrophy and intramedullary T2 weighted imaging (T2WI) hyperintensity were evaluated on lateral and axial MRI. Data were compared between the EN and EN groups or FI and FN groups with T-tests, chi-square tests or Kruskal-Wallis tests. Prediction criteria were determined with logistic regression analyses.

**Results:**

Forty-nine patients were included, and 9 (18.4%) and 11 (22.4%) showed improved extension and flexion SSEPs compared to their own neutral SSEPs, respectively. Interestingly, EI or FI patients had significantly better one-year postoperative mJOA recoveries than EN or FN patients (T-test, *P* < 0.001). Moreover, the disease duration (T-test, *P* = 0.024), involved segment number (Kruskal-Wallis test, P < 0.001), and cervical alignment type (chi-square test, *P* = 0.005) varied significantly between the EI and EN groups. The FI group presented a significantly higher Mühle stenosis grade than the FN group (Kruskal-Wallis test, *P* = 0.038). Furthermore, ≤ 2 involved segments and straight or sigmoid cervical alignment were significant criteria predicting improved extension SSEPs (probability: 85.7%), while Mühle stenosis Grade 3 and disease duration ≤6 months were significant criteria predicting improved flexion SSEPs (probability: 85.7%).

**Conclusions:**

Our findings provide evidence for neurophysiological improvement in patients with DCM at extension and flexion and its significance in predicting prognoses. Moreover, certain clinical and radiographic criteria may help predict neurophysiological improvement upon extension or flexion.

**Trial registration:**

“[2020]151”. Retrospectively registered on April 30, 2020.

## Introduction

Static and dynamic narrowing of the cervical canal is one of the most important factors that causes degenerative cervical myelopathy (DCM) [1]. Cervical motions might rapidly alter (improve or worsen) cervical and referred symptoms, depending on the direction of end-range positioning of the cervical spine [2]. Several studies have investigated dynamic magnetic resonance imaging (MRI) changes in the cervical spine of patients with DCM and have suggested that extension causes narrowing of the spinal canal due to the pincer effect, while flexion longitudinally stretches the cord, both of which exacerbate spinal cord impingement [3]. Somatosensory evoked potentials (SSEPs) have been utilized as useful neurophysiological indicators of objective functional abnormalities of the spinal cord [4, 5]. Based on these studies, we developed dynamic somatosensory evoked potentials (DSSEPs), which are performed at neutral, extension, and flexion positions to evaluate patients’ neurophysiological changes at different neck positions [6]. We reported that most patients presented deteriorating DSSEPs upon extension and flexion [7, 8]. For those patients, their DSSEP N13 amplitude ratios correlated with their preoperative symptomatic severities, postoperative recovery rates, and degrees of radiographic spinal cord compression [8]. However, some patients with DCM presented significantly improved DSSEPs during cervical flexion and/or extension, whose DSSEP N13 amplitude ratios were obviously unmatched to their MRI compression degrees [7, 8]. Along with improved neural electrophysiological results, these patients also frequently presented symptomatic alleviations at extension or flexion.

Currently, few studies have reported extension and flexion neurophysiological functional improvements in DCM cases. Based on our previous finding of the correlations between DSSEPs and radiographic results [8], we expanded our sample size and sorted patients with improved DSSEPs at extension or flexion from other patients to investigate their common preoperative clinical and radiographic characteristics. We also investigated the differences in the prognoses between patients with or without DSSEP improvements and determined criteria for predicting extension- or flexion-induced DSSEP improvements with clinical and neutral position imaging.

## Methods

### Patient cohort

The study was performed in accordance with the Declaration of Helsinki and was retrospectively registered on April 30, 2020 (Trial registration number: [2020]151). We used only medical data recorded for consecutive patients with DCM who were treated at our department and had completed preoperative DSSEPs and MRI tests between 2015 and 2019. The institutional review board (IEC) for clinical and animal trials of the First Affiliated Hospital of Sun Yat-sen University approved the study and waived the requirement for informed consent. All participants received decompression surgeries and were followed for at least 1 year. Patients with a previous surgical or trauma history, spinal tumor, or peripheral neurological disease were excluded. The demographic data collected included sex, age and critical comorbidities. Thirty-eight subjects overlapped with our previous report [8], which evaluated correlations between amplitude ratios of DSSEPs and MRI measurements. DCM disease duration (i.e., time from the onset of DCM-related neurological signs) and clinical signs, including gait impairment, upper limb weakness and the Hoffmann sign, were documented. The mJOA scores [9] for each patient at the time of DSSEP tests and one year postsurgery were recorded. A change in mJOA (ΔmJOA) was computed between baseline and one year postoperatively to evaluate neurological outcomes.

### Realization and measurement of DSSEPs

An electrophysiological monitoring system (Nicolet Endeavor CR) was used to elicit and record the DSSEPs. Median and ulnar nerve DSSEPs were examined using established methods described in our previous study [6, 8]. Recording electrodes were placed over the spinous process of the 2nd cervical vertebra (C2S), the contralateral parietal cortex (Cc) and forehead reference site (Fz) regions of the scalp, and Erb’s points ipsilateral (EPi) and contralateral (EPc) to the stimulation electrodes [10]. The DSSEP waves for each recording montage, which were labeled EPi-EPc, C2S-EPc, and Cc-Fz, were recorded as N9, N13, and N20, respectively. We adopted N9 as the standard reference channel. When N9 was unidentifiable or poorly reproducible, the existence of peripheral nerve pathology was suspected. The DSSEPs were first measured at a neutral neck position. Patients were then tested with neck positions at approximately 35° flexion followed by approximately 20° extension of the cervical spine using a device for elevating the head and neck with minimal discomfort to the subject. Each measurement was performed at least three times by a spine surgeon and two electrophysiologists to confirm the reproducibility of the DSSEPs.

**Fig. 1:**
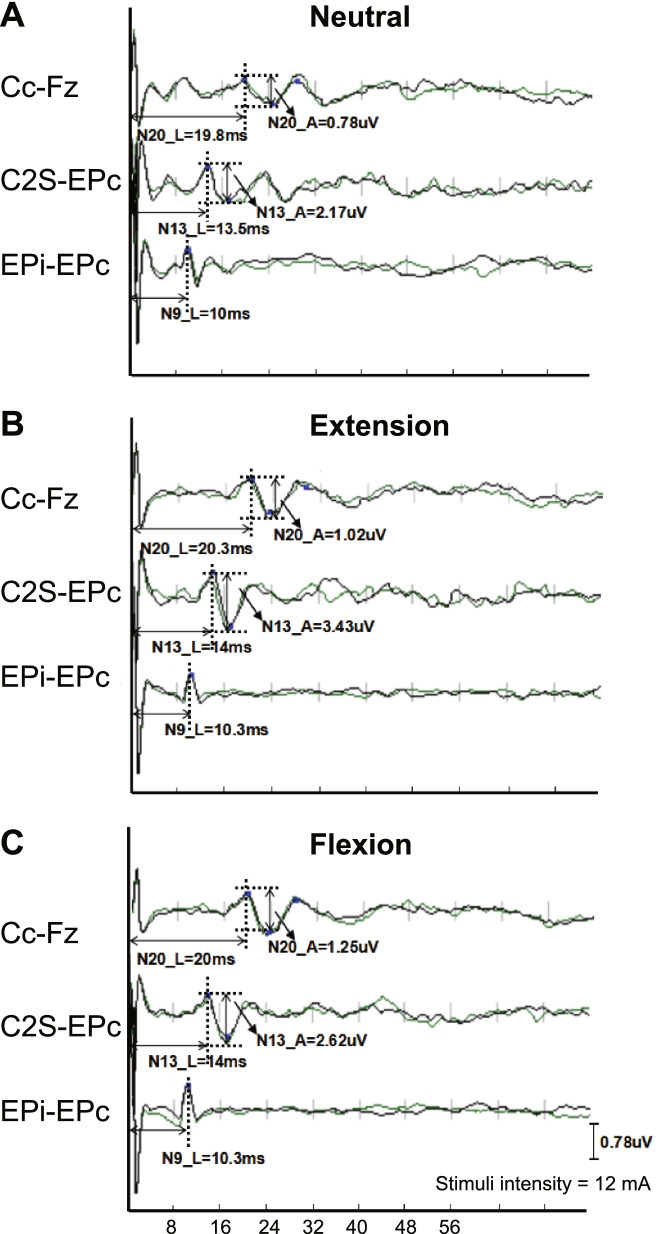
DSSEPs results from a 51-year-old male in the EI/FI group. The DSSEPs were performed at cervical neutral (**A**), 20° extension (**B**) and 35° flexion (**C**) positions for a patient. The latencies of N9, N13 and N20 waves and the amplitudes of N13 and N20 waves are shown in the figure. The patient’s DSSEP N13 amplitude improved by 58.1 and 20.7% at extension and flexion positions respectively compared to the neutral position, meeting the criteria for entering both EI and FI groups (> 20%)

We compared the same patient’s median nerve SSEP upon extension or flexion with those in the neutral position. An improvement in the DSSEP upon extension or flexion was defined as a shorter N13 or N20 latency exceeding 2.5 SD of that at neutral position (which were 1.78 ms for N13 and 2.01 ms for N20 in this study); or increased N13 or N20 amplitude exceeding 20% compared with the patient’s SSEP in the neutral position (Fig. 1). We defined an immeasurable SSEP as a waveform that was not able to be identified by averaging over 500 sweeps. Any measurable SSEP waveform would be considered an improved DSSEP compared with an immeasurable SSEP waveform. Patients with improved DSSEPs upon extension or flexion were classified into the extension-improved (EI) or flexion-improved (FI) groups respectively. Otherwise, the patients were classified into the extension-nonimproved (EN) or flexion-nonimproved (FN) group.

### Imaging methods and analytical protocol

All MR examinations were performed with a 3.0-T MR imager (Siemens Trio) with the patients lying in the supine position using a spine-array coil. The authors evaluated compressed spinal cords by capturing standard imaging sequences.

Qualitative MRI features on sagittal T2-weighted sequences included the presence of cervical ligamentum flavum hypertrophy (LFH) and spinal cord intramedullary T2WI signal hyperintensity (IHI). LFH is defined as a thickened ligamentum flavum compared with the thickness of adjacent segments, along with a loss of epidural fat tissue and dural sac compression [11].

Quantitative MRI features on T2-weighted sequences included the number of stenotic segments, cervical stenosis grade (classification described by Mühle et al. [12], Grades 0 to 3) and disc degeneration grade (classification described by Miyazaki et al. [13], Grades 1 to 5) of the most compressed segment.

**Fig. 2:**
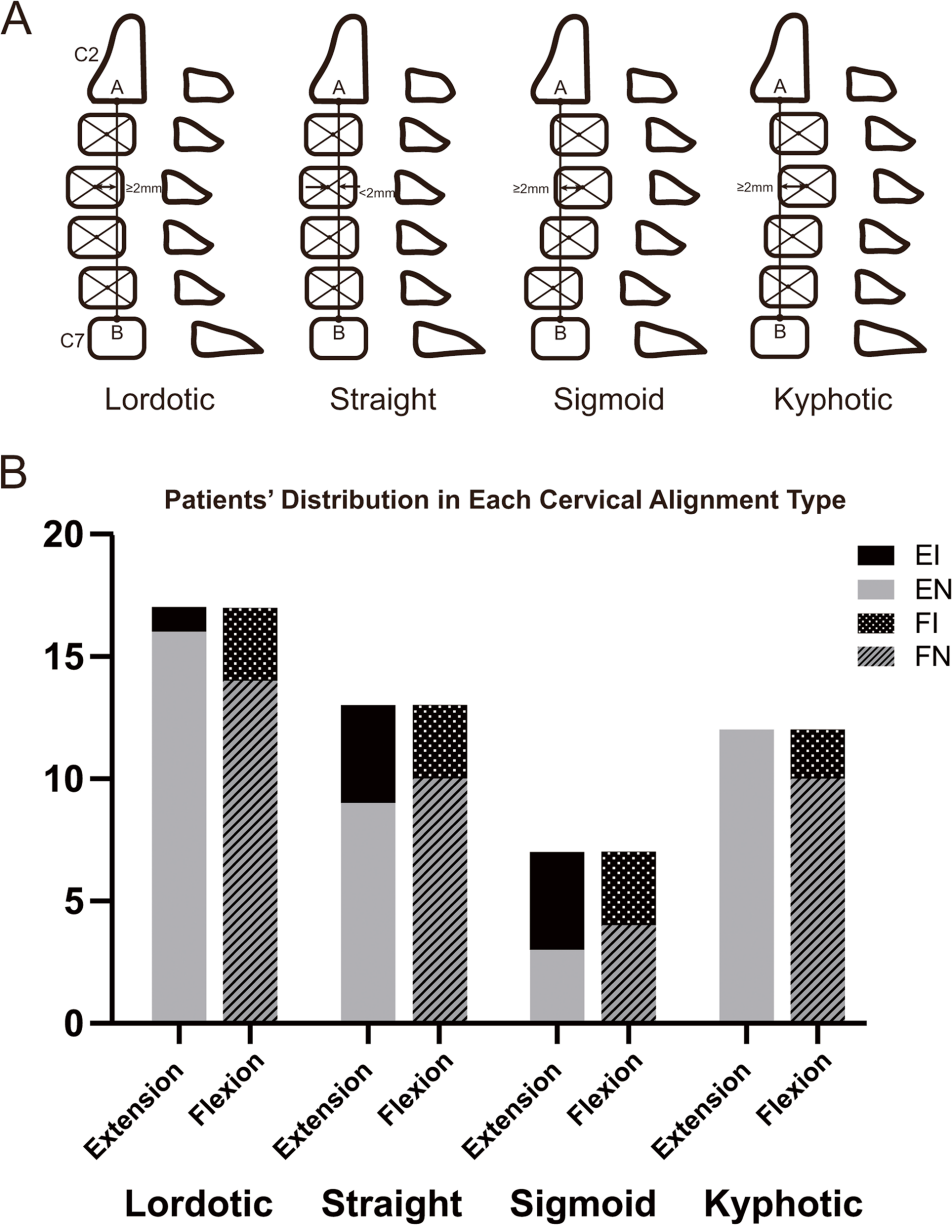
**A **Cervical alignment types (modified Toyama method [16]): A line connecting the midpoints of the inferior margin of C2 and the superior margin of C7 was constructed. Lordotic group: all centroids are anterior to the line and the distance between at least one centroid and the line is ≥2 mm; Straight group: the distance between the line and each centroid is less than 2 mm; Sigmoid group: some centroids are anterior to and some posterior to the line and the distance between the line and at least one centroid is ≥2 mm; Kyphotic group: all the centroids are posterior to the line and the distance between at least one centroid and the line is ≥2 mm. **B **The distribution of patients with DCM presenting different changes in DSSEPs in each cervical alignment type

The spondylolisthesis group was defined as those with ≥2 mm of slippage, which was measured as the distance from the posteroinferior corner of the cranial vertebral body to the tangential line along the posterior border of the caudal vertebral body, and the direction of spondylolisthesis was recorded as anterolisthesis or retrolisthesis based on the level with the highest grade on the plain lateral radiographs [14, 15]. The cervical alignment types were also measured on plain cervical lateral radiographs and categorized into one of the following four groups using the modified Toyama method [16] (Fig. 2A).

These measurements were carried out by 3 independent investigators.

### Statistical analysis

Differences in age, DCM disease duration, mJOA and ΔmJOA scores between the EI/FI and EN/FN groups were calculated with Student’s T-test. The differences in DSSEP changes recorded upon flexion, cervical alignment types, ligamentum flavum hypertrophy and intramedullary intensity between the EI/FI and EN/FN groups were calculated with chi-square tests. The numbers of stenotic segments, Mühle stenosis grade and disc degeneration stage were compared between these groups with the Kruskal-Wallis test.

A bivariate logistic analysis was used to evaluate the relationship between the improvement in DSSEPs upon extension or flexion and a set of clinical and radiographic criteria of interest. Variables with *p* < 0.2 in the bivariate analysis were entered into a forward stepwise multivariate logistic regression model [17]. Model fit was assessed with the omnibus tests of model coefficients and Hosmer-Lemeshow goodness-of-fit test. A significant value for the omnibus chi-square test indicates a credible improvement of the new model over the baseline model, and a nonsignificant value for the Hosmer-Lemeshow chi-square test suggests an absence of biased fit. After the final logistic model was established, the probability of an improved DSSEP upon extension or flexion was calculated. The statistical software R (R version 3.6.1) was used for statistical analyses.

## Results

### Demographic and clinical results for patients in each group

Fifty-six consecutive surgically treated patients with DCM were primarily enrolled, and 49 (55.8 ± 11.3 years; 28 men) were finally included in this study. Seven patients were excluded because of histories of cervical spine surgeries, diabetic peripheral neuropathy, or median nerve injury. Among the 49 patients, 9 (18.4%) exhibited improvements in DSSEPs upon extension, and 11 (22.4%) had DSSEPs that improved upon flexion. All 9 EI patients underwent the anterior cervical discectomy and fusion (ACDF) procedure, while 8, 1, 2 FI patients underwent ACDF, posterior and antero-posterior combined surgeries respectively. The preoperative and one-year postoperative mJOA scores for all patients were 14.84 ± 1.78 and 16.84 ± 2.07, respectively. Although no differences in the preoperative and postoperative mJOA scores were observed between the EI and EN groups or the FI and FN groups, the ΔmJOA of the EI/FI group was significantly higher than that of the EN/FN group (T-test, *p* < 0.001). In addition, patients in the EI group had a significantly shorter disease duration (T-test, *p* = 0.024) than patients in the EN group. No differences in sex, age, gait impairment, upper limb weakness, or positive Hoffmann signs were observed between the EI and EN groups. No differences were found for any other demographic or clinical data apart from ΔmJOA between the FI and FN groups (Table 1).

**Table 1:** Comparison of each group with respect to demographic, clinical, and radiographic features

Group	DSSEP change upon extension			DSSEP change upon flexion			Statistical method
	Extension-Improved (EI)	Extension-Non-improved (EN)	P	Flexion-Improved (FI)	Flexion-Non-improved (FN)	P	
No.(%)	9 (18.4%)	40 (81.6%)		11 (22.4%)	38 (77.6%)		
Sex male (female)	3 (6)	25 (15)	0.221	5 (6)	23 (15)	0.587	Chi-square
Age (mean ± SD)	58.56 ± 12.77	55.2 ± 11.27	0.445	59.91 ± 10.88	54.63 ± 11.57	0.193	T-test
Preoperative Clinical Assessments							
CSM disease duration (months)	11.44 ± 10.57	30.28 ± 29.30	0.024*	15.82 ± 16.79	30.01 ± 29.49	0.142	T-test
Gait impairment Yes (No)	4 (5)	22 (18)	0.295	8 (3)	18 (20)	0.254	Chi-square
Upper limb weakness Yes (No)	4 (5)	22 (18)	0.719	6 (5)	20 (18)	1	Chi-square
Hoffmann sign (Yes/No)	6 (3)	12 (28)	0.425	8 (3)	16 (22)	0.148	Chi-square
Preoperative mJOA score	14.56 ± 1.50	14.9 ± 1.83	0.565	14.09 ± 1.50	15.05 ± 1.79	0.119	T-test
Preoperative Radiological Assessments							
Cervical Spondylolisthesis (Non/Anterolisthesis/Retrolisthesis)	7/0/2	22/2/16	0.422	7/0/4	22/2/14	0.731	Chi-square
Cervical alignment (Lordosis/Straight/ Sigmoid/Kyphosis)	1/4/4/0	16/9/3/12	0.005**	3/3/3/2	14/10/4/10	0.545	Chi-square
Ligamentum flavum hypertrophy (Yes/No)	3 (6)	27 (13)	0.128	7 (4)	23 (15)	1	Chi-square
Intramedullary T2WI hyperintensity (Yes/No)	4 (5)	30 (10)	0.163	7 (4)	27 (11)	0.922	Chi-square
No. Involved segments (mean ± SD)	1.56 ± 0.68	2.95 ± 1.09	< 0.001***	2.27 ± 1.05	2.82 ± 1.17	0.269	Kruskal-Wallis
Disc degeneration grade (mean ± SD)	3.56 ± 1.17	4.13 ± 0.90	0.182	3.91 ± 1.08	4.05 ± 0.94	0.752	Kruskal-Wallis
Mühle stenosis grade (mean ± SD)	2.22 ± 0.79	2.2 ± 0.75	0.938	2.64 ± 0.64	2.08 ± 0.74	0.031*	Kruskal-Wallis
No. patients undergoing each surgical procedure							
Anterior/Posterior/ Combined	9/0/0	29/7/4	0.203	8/1/2	30/6/2	0.358	Chi-square
One-year Postoperative Clinical Assessment							
mJOA score	17.67 ± 1.41	16.65 ± 2.15	0.191	17 ± 2.04	16.79 ± 2.08	0.773	T-test
ΔmJOA score	3.11 ± 0.57	1.75 ± 0.8	< 0.001***	2.91 ± 0.79	1.74 ± 0.78	< 0.001***	T-test

* *p* < 0.05; ** *p* < 0.01; *** *p* < 0.001

*DSSEP* dynamic somatosensory evoked potential

### Comparison of the radiographic characteristics of each group

Nineteen patients presented cervical spondylolisthesis. There were 17, 13, 7 and 12 patients presented cervical lordotic, straight, sigmoid and kyphotic cervical alignments, respectively. A total of 132 stenotic segments were identified in all 49 patients with DCM. C5/6 was the most commonly affected segment (*n* = 46), followed by the C4/5 segment (*n* = 37). Compared with patients in the EN group, patients in the EI group were more likely to present straight or sigmoid cervical alignment (chi-squared, *p* = 0.005) (Fig. 2B) and had significantly fewer stenotic segments (Kruskal-Wallis, *p* < 0.001). The EI group also tended to have absent LFH (chi-square, *p* = 0.128) and IHI (chi-square, *p* = 0.163) and a lower disc degeneration grade (Kruskal-Wallis, *p* = 0.182), although the differences were not statistically significant. Patients in the FI group had significantly more severe Mühle stenosis grades (Kruskal-Wallis, *p* = 0.038) than those in the FN group. Two and 18 patients presented anterolisthesis and retrolisthesis, respectively. No differences in cervical spondylolisthesis types were observed between the EI and EN or FI and FN groups. More detailed data are shown in Table 1.

### Dichotomous classification based on clinical and radiographic results and forward stepwise multivariable regression analysis for predicting improved DSSEPs upon extension and flexion

Based on the findings described above, we selected clinical and radiographic indicators of potential significance that might predict improved DSSEPs upon extension and divided patients into groups according to the following 6 dichotomous criteria: disease duration ≤6 months, stenotic levels ≤2, straight or sigmoid cervical alignment, disc degeneration grade ≤ 3, absence of LFH, and absence of IHI. All 6 criteria met the entrance criteria into our multivariate logistic regression model for predicting improved DSSEPs at extension (Logit *P* < 0.2). Similarly, we divided patients into groups according to the following 4 potentially significant dichotomous criteria for predicting improved DSSEPs at flexion: disease duration ≤6 months, mJOA score < 15, positive Hoffmann sign, and Mühle Grade 3 stenosis. Except for the presence of the Hoffmann sign, the remaining 3 criteria were significant in our primary binary logistic regression model (Logit *P* < 0.2) and were input into the multivariable logistic regression model for predicting improved DSSEPs at flexion (Table 2).

**Table 2:** Chi-square and primary binary logistic regression analysis of dichotomous criteria for DSSEP improvement upon extension and flexion

DSSEP change upon extension							
	EI Group Yes (No)	EN Group Yes (No)	χ2 *p* value	Logit Coefficient B	Standard Error	Logit *P* Value	Odds Ratio (95% CI)
Disease Duration ≤6 months	6 (3)	9 (31)	0.028*	1.93	0.802	0.016^†^	6.889 (1.43–33.182)
Stenotic segment number ≤ 2	8 (1)	14 (26)	0.0103*	2.698	1.111	0.015^†^	14.857 (1.683–131.171)
Straight or sigmoid alignment	8 (1)	12 (28)	0.004**	2.927	1.115	0.009^†^	18.667 (2.097–166.139)
Disc degeneration grade ≤ 3	5 (4)	10 (30)	0.163	1.322	0.764	0.084^†^	0.267 (0.06–1.191)
Absence of LFH	6 (3)	13 (27)	0.128	1.424	0.784	0.069^†^	0.241 (0.052–1.118)
Absence of IHI	5 (4)	10 (30)	0.163	1.322	0.764	0.084^†^	0.267 (0.06–1.191)
DSSEP change upon flexion							
	FI Group Yes (No)	FN Group Yes (No)	χ2 *p* value	Logit Coefficient B	Standard Error	Logit *P* Value	Odds Ratio (95% CI)
Disease duration ≤6 months	6 (5)	9 (29)	0.113	1.352	0.716	0.059^†^	3.867 (0.951–15.724)
mJOA < 15	7 (4)	14 (24)	0.217	1.099	0.711	0.122^†^	0.333 (0.083–1.344)
Hoffmann sign	8 (3)	16 (22)	0.148	0.771	0.707	0.275	2.162 (0.541–8.635)
Mühle grade 3	8 (3)	13 (25)	0.054	1.635	0.758	0.031^†^	5.128 (1.160–22.676)

*, **: Chi-square *p* < 0.05, *p* < 0.01

^†^ Variables with *p* < 0.2 in the bivariate analysis were entered into the forward stepwise multivariate logistic regression models

*DSSEP* dynamic somatosensory evoked potential

The final multivariate regression model showed that the presence of cervical spondylolisthesis (OR = 20.26, *P* = 0.019) and straight or sigmoid cervical alignment (OR = 32.071, *P* = 0.017) were significant criteria for predicting improved DSSEPs upon extension. The three-step forward regression model (omnibus test, χ^2^ = 25.291, *P* < 0.001; Hosmer-Lemeshow test, χ^2^ = 4.975, *P* = 0.419) predicted 93.9% of the cases. Mühle Grade 3 stenosis (OR = 7.295, *P* = 0.017) and disease duration ≤6 months (OR = 5.165, *P* = 0.044) were significant criteria predicting improved DSSEPs upon flexion in our two-step forward regression model (omnibus test, χ^2^ = 10.321, *P* = 0.006; Hosmer-Lemeshow test, χ^2^ = 2.619, *P* = 0.270) and predicted 85.7% of the cases (Table 3).

**Table 3:** Final forward stepwise multiple linear regression model relating the best combination of clinical and imaging predictors to the DSSEP improvement upon extension and flexion

	Variables	Logit Coefficient B	Standard Error	*P* Value	Odds Ratio	Predicted Probability
DSSEP improvement upon Extension						
Step 1	With straight or sigmoid alignment	2.927	1.115	0.009*	18.667	81.6%
	Constant	−3.332	1.018	0.001	0.036	
Step 2	Stenotic segment number ≤ 2	2.508	1.174	0.033*	12.275	85.7%
	With straight or sigmoid alignment	2.754	1.165	0.018*	15.711	
	Constant	−4.876	1.438	0.001	0.008	
Step 3	Stenotic segment number ≤ 2	2.905	1.373	0.034*	18.272	85.7%
	With straight or sigmoid alignment	3.341	1.402	0.017*	28.253	
	Disc degeneration grade ≤ 3	2.368	1.251	0.058	10.677	
	Constant	−6.478	2.083	0.002	0.002	
DSSEP improvement upon Flexion						
Step 1	Mühle grade 3	1.754	0.762	0.021*	5.778	77.6%
	Constant	−2.159	0.61	0	0.115	
Step 2	Disease duration ≤6 months	1.642	0.816	0.044*	5.165	85.7%
	Mühle grade 3	1.987	0.834	0.017*	7.295	
	Constant	−2.914	0.804	0	0.054	

* *p* < 0.05

## Discussion

DSSEPs are sensitive tools to evaluate DCM patients’ neurophysiological changes at dynamic neck positions [6], which are correlated with preoperative clinical severities and MRI compression degrees and IHI signal features [8]. In most DCM populations, dynamic neck postures cause DSSEPs deteriorations as demonstrated by decreased N13 and N20 amplitudes and prolonged latencies in previous dynamic electrophysiological studies [6, 8, 18]. These results correspond with most dynamic MRI studies, that cervical extensions make the ligamentum flavum bulge inward, decrease the dorsal subarachnoid space up to 17% [19] and increase the Mühle stenosis grade [20], while cervical flexion increases the longitudinal strain of the spinal cord and induces compression against the ventral spondylotic bar [21]. Because of these, prolonged extension and flexion, especially extension positions, are commonly recognized as deleterious activities for patients with DCM [22]. To the best of our knowledge, this cohort study is the first to report neurophysiological improvements among patients with DCM upon dynamic positioning and to evaluate its prognostic value and identify the clinical and radiographic factors related to neurological improvements observed upon cervical extension and flexion.

We reported 9 (18.4%) and 11 (22.4%) DCM patients exhibited significant improvement upon extension and flexion in the current study. Interestingly, many patients in the EI group reported their preference for activities requiring neck extension, such as badminton and some types of gymnastics, whereas patients in the FI group usually felt more comfortable at flexion, suggesting consistency between symptomatic and DSSEP changes and indicating that the extended and flexed positions might relieve patients’ neurological deficits in some cases [23]. The EI group patients’ neurophysiological improvement at cervical extension could be explained by the decreased longitudinal length and stretching force of the cervical cord shown in extension MRI [24]. Upon cervical flexion, the dorsal subarachnoid space increases at each level from C2 to C7 [19], leading to decompression and functional improvement of the cord in some DCM patients in FI group. Based on these results, cervical extension and flexion do not always cause neurophysiological deteriorations.

All patient in the EI group underwent an ACDF procedure, which is an effective method for recovering a physiological lordotic cervical alignment [25]. Three FI patients underwent posterior or antero-posterior surgeries, which are mainly used in DCM patients with multi-segmental or severe antero-posterior spinal cord compression [25]. Despite the variations in decompressive surgical methods, the one-year postoperative ΔmJOA score of both of the EI and FI groups were significantly higher than that of the EN and FN groups respectively, indicating better recuperation capacities of patients exhibiting improved DSSEPs at extension and/or flexion. Age, duration of symptoms and baseline mJOA score were reported to be significant predictors of postoperative outcomes in patients with DCM in some other studies [26]. In the present study, although patients in the EI group exhibited no differences in age, sex, mJOA scores, or several other clinical signs and symptoms compared with those in the EN group, they had significantly shorter disease durations. A disease duration ≤6 months was also found to be a significant predictive criterion for improved DSSEPs upon flexion in the present study. A potential explanation for this finding is that newly impinged spinal cords generally exhibit greater neurological preservation and are more easily reversible than those of patients suffering from long-standing compression at dynamic neck positions.

**Fig. 3:**
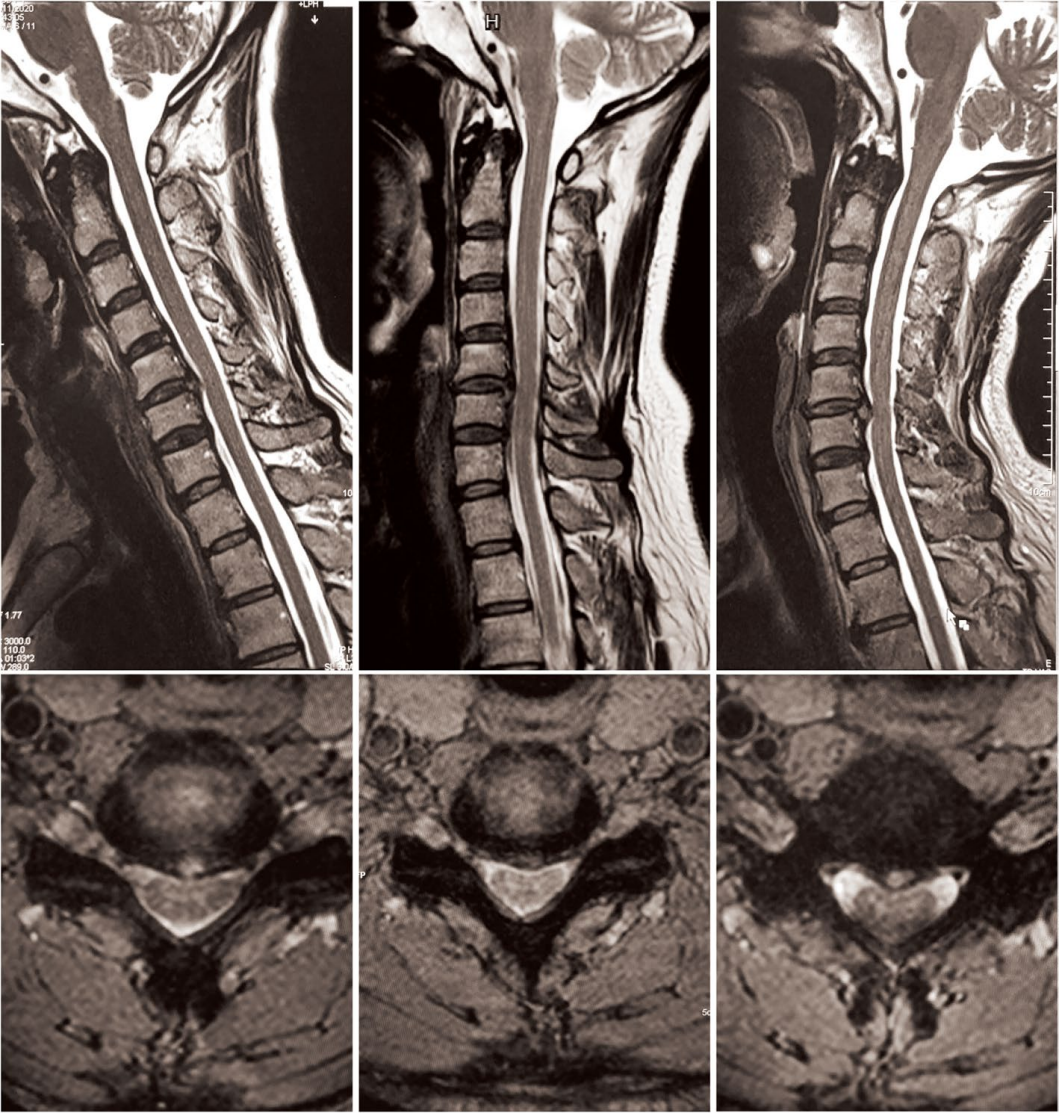
Dynamic MR images of a patient with improved DSSEPs at extension and deteriorated DSSEPs at flexion. Panels from left to right show cervical flexion, neutral and extension positions. Upon neutral positioning, this patient had a straight cervical alignment and a single protruding C5/6 segment. The Mühle stenosis grade of this patient was Grade 1. Upon flexion, the spinal cord was longitudinally stretched and draped backward. The cerebral fluid in front of the spinal cord was narrower in the axial image. Upon extension, although the diameter of his cervical canal did not change significantly, the spinal cord was longitudinally relaxed and draped backward and, therefore, ameliorated anterior compression to some extent. The cerebral fluid in front of the spinal cord was wider in the axial image captured in the extension position

Regarding the radiographic characteristics, we found that the number of involved segments was significantly smaller in the EI group than in the EN group, and ≤ 2 involved segments was a significant criterion for predicting improved DSSEPs at extension. Previous dynamic MRI studies revealed that for patients with multiple involved segments, many of the segments that were not significantly compressed in the neutral position narrowed substantially upon extension [1, 24, 27]. Thus, patients with multilevel stenosis would suffer more serious neurological deterioration upon extension, probably due to significantly less compensative space resulting from multiple segmental pincer effects, as shown in our dynamic MRI of a patient with deteriorated flexion DSSEPs. In contrast, fewer segments usually cause focal and limited compression and leave more compensatory space, allowing the patient’s neurological deficits to be more easily relieved upon extension. EI patients also tended to have straight or sigmoid cervical alignments, which is another significant criterion for predicting improved DSSEPs upon extension. For patients with DCM presenting with either of the two alignment types, their cervical cords were usually tightly longitudinally stretched and suffered from focal anterior compression, such as protruding discs or osteophytes from focal kyphosis in the neutral position. During extension, their cervical cords would be longitudinally relaxed and draped backward, thus ameliorating the stretching tension and the anterior compression to some extent [24]. as shown in our dynamic MRI for a patient with improved extension DSSEPs (Fig. 3). Patients with a lordotic alignment will not experience such benefits because their cords are already longitudinally relaxed in their neutral positions [1]. Patients with a kyphotic alignment experienced much more severe potential ligamentum flavum bulges and pincer effects upon extension [28–30]. which might offset the benefits of decreased longitudinal tension.

In addition to a disease duration ≤6 months, Mühle stenosis Grade 3 was another significant criterion for predicting improved DSSEPs upon flexion. Based on accumulating evidence, patients with DCM have expanded cervical canals, even with cord decompression on flexion MRI [3, 24, 27, 31]. The diameter of the dorsal subarachnoid space at each level from C2 to C7 might increase up to 89% in flexion [19]. According to those dynamic MRI results, the severely compressed spinal cord of patients with Muhle Grade 3 stenosis on neutral MRI would probably experience greater benefits from spinal canal enlargement upon flexion, and thus these patients are more likely to present improved DSSEPs upon flexion. Cervical spondylolisthesis potentially lead to narrowing of the cervical canal and increased translational motion, which by itself might exacerbate myelopathy [32]. Spondylolisthesis at C2/3 and C7/T1 is also reported to be associated with worse mJOA scores upon extension and flexion in patients with DCM [33]. In our study, most patients with spondylolisthesis exhibited deteriorated DSSEP sat both extension and flexion, but the correlation between spondylolisthesis and the changes in DSSEPs was not significant.

The study has a few limitations. First, this is a retrospective study characterized by low-level evidence, which is a notable limitation. Second, the sample size of the study was relatively small, which limited statistical power. Besides, the lack of comprehensive dynamic MRI data in this study also undermines its persuasiveness. A prospectively designed study with larger sample size and dynamic MRI data is warranted to address these problems. Lastly, although the test has been proven safe and effective for DCM diagnosis and evaluation [7, 8], the dynamic neck flexion and extension positions might be harmful to certain patients with severe myelopathy. Thus, physicians should carefully evaluate patients’ condition and notify the risks and benefits to the patients before the examination, and discontinue immediately once the patient complain of severe discomfort or exacerbating paresthesia.

## Conclusions

Our findings provide evidence for neurophysiological improvement in patients with DCM at different neck positions and its significance in predicting prognoses. Furthermore, several preoperative clinical and radiographic results, such as ≤2 involved segments and straight or sigmoid cervical alignment, were significant predictors of improved DSSEPs upon extension, while Mühle stenosis Grade 3 and a disease duration ≤6 months were significant predictors of improved DSSEPs upon flexion.

## Data Availability

The datasets generated during the current study are available from the corresponding author upon reasonable request.

## Abbreviations

DCM: Degenerative cervical myelopathy
DSSEP: Dynamic somatosensory evoked potential
SSEP: Somatosensory evoked potential
MRI: Magnetic resonance imaging
CR: Compression ratios at the compressed site in MRI axial images
EI: DSSEP extension-improved group
EN: DSSEP extension-nonmproved group
FI: DSSEP flexion-improved group
FN: DSSEP flexion-nonimproved group
